# Integrated multi-omics analyses reveal Jorunnamycin A as a novel suppressor for muscle-invasive bladder cancer by targeting FASN and TOP1

**DOI:** 10.1186/s12967-023-04400-3

**Published:** 2023-08-16

**Authors:** Ruijiao Chen, Xiaopeng Hao, Jingyuan Chen, Changyue Zhang, Huixia Fan, Fuming Lian, Xiaochuan Chen, Chao Wang, Yong Xia

**Affiliations:** 1 Medical Laboratory of Jining Medical University, Jining Medical University, Jining, 272067 Shandong China; 2 Institute of Precision Medicine, Jining Medical University, No. 133 Hehua Road, Taibaihu District, Jining, 272067 Shandong China; 3 School of Pharmacy, Binzhou Medical University, Yantai, 264003 Shandong China; 4 Key Laboratory of Green Chemistry and Technology of Ministry of Education, College of Chemistry, Sichuan University, Chengdu, 610064 China; 5 Department of Urology, Jining No. 1 People’s Hospital, Jining, 272106 Shandong China

**Keywords:** Jorunnamycin A, Muscle invasive bladder cancer, Proteomics, Transcriptomics, Proliferation, Apoptosis, Molecular docking

## Abstract

**Background:**

Bladder cancer is a urological carcinoma with high incidence, among which muscle invasive bladder cancer (MIBC) is a malignant carcinoma with high mortality. There is an urgent need to develop new drugs with low toxicity and high efficiency for MIBC because existing medication has defects, such as high toxicity, poor efficacy, and side effects. Jorunnamycin A (JorA), a natural marine compound, has been found to have a high efficiency anticancer effect, but its anticancer function and mechanism on bladder cancer have not been studied.

**Methods:**

To examine the anticancer effect of JorA on MIBC, Cell Counting Kit 8, EdU staining, and colony formation analyses were performed. Moreover, a xenograft mouse model was used to verify the anticancer effect in vivo. To investigate the pharmacological mechanism of JorA, high-throughput quantitative proteomics, transcriptomics, RT-qPCR, western blotting, immunofluorescence staining, flow cytometry, pulldown assays, and molecular docking were performed.

**Results:**

JorA inhibited the proliferation of MIBC cells, and the IC_50_ of T24 and UM-UC-3 was 0.054 and 0.084 μM, respectively. JorA-induced significantly changed proteins were enriched in “cancer-related pathways” and “EGFR-related signaling pathways”, which mainly manifested by inhibiting cell proliferation and promoting cell apoptosis. Specifically, JorA dampened the DNA synthesis rate, induced phosphatidylserine eversion, and inhibited cell migration. Furthermore, it was discovered that fatty acid synthase (FASN) and topoisomerase 1 (TOP1) are the JorA interaction proteins. Using DockThor software, the 3D docking structures of JorA binding to FASN and TOP1 were obtained (the binding affinities were − 8.153 and − 7.264 kcal/mol, respectively).

**Conclusions:**

The marine compound JorA was discovered to have a specific inhibitory effect on MIBC, and its potential pharmacological mechanism was revealed for the first time. This discovery makes an important contribution to the development of new high efficiency and low toxicity drugs for bladder cancer therapy.

**Graphical Abstract:**

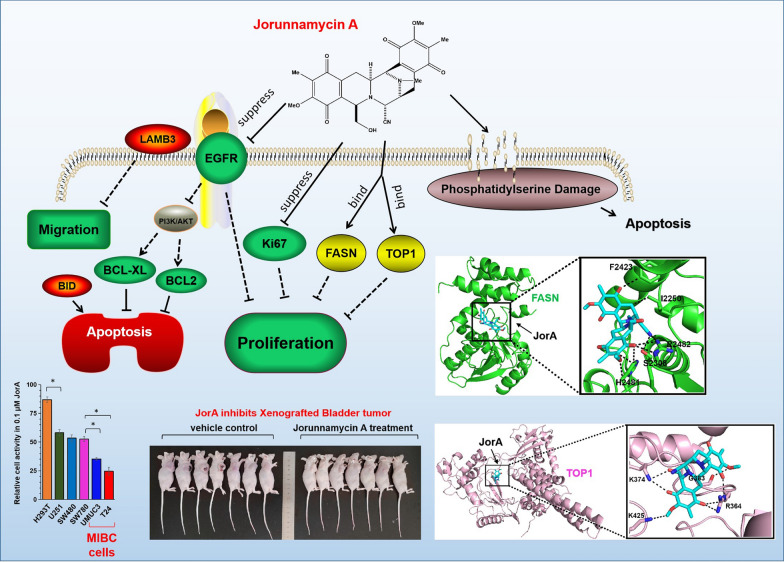

**Supplementary Information:**

The online version contains supplementary material available at 10.1186/s12967-023-04400-3.

## Background

Bladder cancer is a serious cancer disease that mainly affects men and elderly people. Bladder cancer seriously impacts human life and health, with almost 170,000 people dying as a result worldwide each year [1, 2]. According to the diagnosis results of different clinical examinations, bladder cancer can be divided into muscle invasive bladder cancer (MIBC) and non-MIBC (NMIBC) [1]. MIBC involves tumors that can infiltrate the bladder muscle layer and tend to spread to other organs. MIBC accounts for about 30% of newly diagnosed bladder cancer cases, and about 15–20% of NMIBC cases can progress to MIBC. The 5-year overall survival rate of patients with MIBC is only 40–50% [3]. However, due to the invasive and metastatic characteristics of MIBC, patients often have to return to the clinic and require surgical resection when they relapse. Therefore, MIBC is one of the worst malignant tumors with high economic burden and health risks.

At present, the clinical treatment of MIBC is usually radical cystectomy and drug chemotherapy [4]. However, radical cystectomy brings huge inconvenience to patients and affects their quality of life. Therefore, patients with MIBC who want to avoid radical cystectomy need more effective drug chemotherapy [5]. Neoadjuvant chemotherapy based on cisplatin is the mainstream choice of chemotherapy at present. However, because MIBC has a strong tendency of distal metastasis leading to recurrence, the cure rate of existing chemotherapy drugs is limited. In addition, due to the lack of consensus on the eligibility criteria for cisplatin, it remains doubtful whether cisplatin is an optimum treatment for patients with MIBC [6]. Moreover, cisplatin resistance and nephrotoxicity also limit the treatment of MIBC. Therefore, identification of new anti-bladder cancer drugs to meet the needs of different patients with bladder cancer is urgently needed.

Jorunnamycin A (JorA), which is found in Thai Blue Sponge (*Xestospongia *sp.), has been reported to have cytotoxicity to human lung cancer cells [7]. It has also been reported that JorA can inhibit epithelial-to-mesenchymal (EMT) transition and sensitize anoikis in human lung cancer cells [8]. A previous study reported that JorA inhibited the stem cell-like phenotype of human lung cancer stem cell-like cells and enhanced cisplatin-induced apoptosis [9]. However, the anticancer activity and pharmacological mechanism of JorA on bladder cancer remain unclear. In this study, the cytotoxicity of JorA to human normal 293 T cells, human glioma cells, colorectal cancer cells, and bladder cancer cells was compared, and it was found that JorA had low cytotoxicity to normal cells, but had obvious cytotoxicity to cancer cells. In particular, MIBC cells showed highest sensitivity to JorA, suggesting that JorA could be a powerful drug for MIBC treatment.

The effects of JorA on mRNAs and proteins in bladder cancer cells were analyzed by transcriptomics and proteomics, and the network pharmacological molecular mechanism of JorA inhibiting MIBC cells was revealed. JorA inhibited MIBC cell proliferation, inhibited cell migration, and induced apoptosis by regulating LAMB3, Keap1, Bid, EGFR, Bcl-2, Bcl-XL, and other molecules. Moreover, pulldown assays and computer simulation technology were used to capture the molecules that may directly interact with JorA in bladder cancer cells, such as fatty acid synthase (FASN) and DNA topoisomerase 1 (Top1), which participate in the biological process of JorA inhibiting MIBC cells. However, other possible pathways involved in the pharmacological mechanism were not ruled out. This study expanded the application scenario of JorA in the field of antitumor treatments, which provides a research reference for further investigation into anti-bladder cancer drugs based on the marine compound JorA, and contributes to the development of new MIBC therapeutic strategies.

## Methods

### Reagent and detection kit

JorA was self-synthesized by the authors’ cooperative group according to the method reported in a previous study [10]. RIPA buffer was obtained from Beyotime Biotechnology (P0013B, Beyotime, China). The protease inhibitor cocktail was obtained from MCE (HY-K0010, MCE, China). SDT buffer for proteomic analysis was supplied by Applied Protein Technology (Shanghai, China). ECL for western blotting was obtained from Beyotime Biotechnology (P0018AS, Beyotime, China). The immunohistochemistry (IHC) kit was purchased from Boster (SV0004, Boster, China). The streptavidin magnetic beads for JorA pulldown assays were obtained from Beyotime Biotechnology (P2151, Beyotime, China). All other reagents were purchased from Solarbio (Beijing, China).

### Cell culture

Bladder cancer T24, UM-UC-3, and SW780 cells; colorectal cancer SW480 cells; glioma U251 cells; and human embryo kidney 293 T cells were all sourced from the ATCC (Manassas, VA, USA). T24 cells were cultured in RPMI 1640 medium and UM-UC-3, SW780, SW480, U251, and H293T cells were cultured in DMEM in a 5% CO_2_ humidified incubator at 37 °C. Both RPMI 1640 medium (01-100-1ACS, BI, Israel) and DMEM (06-1055-57-1ACS, BI, Israel) contained 10% fetal bovine serum (10099-141, Gibco, Australia) and 1% penicillin and streptomycin (UB89609, Bioodin, China).

### Cell activity measurement

Cells were treated with JorA in 96-well plates (TCP011096, JETBIOFIL, China). Cell Counting Kit-8 (CCK8; CK04, Dojindo, Japan) was employed to measure cell activity according to the manufacturer’s instructions. The absorbance at 450 nm was read with a microplate reader (Cytation5, BioTek, USA).

### Cell colony formation

MIBC T24 cells were seeded in a 12-well plate with an original cell density of 500 cells per well. When a single cell formed a colony composed of 4–8 cells, different concentrations of JorA were added. The cell culture medium was changed every 3 days (JorA was also renewed). After 14 days, the cells were fixed with 4% paraformaldehyde and stained with crystal violet.

### Cell migration measurement

Cell Culture-Insert 4 Well (#80469, Ibidi, Germany) was used for the cell migration experiment. T24 cells were incubated with JorA and the migration ratio of T24 cells was recorded through a microscope (Nikon, Eclipse Ti-S, Japan).

### DNA replication detection

EdU staining (5-ethynyl-2′-deoxyuridine) was used to test DNA replication. T24 cells treated with JorA were stained with the EdU Cell Proliferation Kit (C0078S, Beyotime, China) according to the manufacturer's instructions. The DNA replication ratio of T24 cells was recorded through the CellInsight CX7 High-Content Screening platform (Thermo Scientific, USA).

### Xenograft experiment

Fourteen 4-week-old Balb/c nude mice were obtained from the Jinan Pengyue Animal Breeding Co., Ltd (Jinan, China). After the mice were raised in the authors’ local animal facility for 1 week, they underwent subcutaneous tumor implantation (6 × 10^6^ T24 cells per mouse). When the tumor size reached 50–100 mm^3^, the mice were divided into two groups: the JorA treatment group and the control group. The JorA treatment group was given JorA (intraperitoneal injection) at a dose of 1 mg/kg (once every 2 days); the control group was given the same dose of solvent (corn oil). At the endpoint, the mice were euthanized with CO_2_, and the tumor, liver, and kidneys were taken for pathological examination. The mouse experiment was approved by the Medical Animal Care & Welfare Committee of Jining Medical University (Approval No. JNMC-2021-DW-003).

### Tandem mass tags-labeled proteomic assay

Tandem mass tags (TMT) technology is a tandem mass spectrometry labeling technology that is mainly used to identify and quantify proteins in different samples. A TMT-labeled proteomic assay can use a variety of stable isotope labels and can compare the relative content of proteins in up to 12 different samples simultaneously. The cells were divided into two groups: the JorA-treated group (JAT) and the control group (CTL), and each group consisted of three samples with 1.5 × 10^7^ T24 cells in each sample. The whole proteins were extracted and stored in SDT buffer (Applied Protein Technology, Shanghai, China). TMT-labeled proteomic assays were performed by Applied Protein Technology (Shanghai, China).

### Immunohistochemical and immunofluorescence experiments

The xenografted tumors were processed into formalin-fixed and paraffin-embedded samples. The samples were sectioned into 4 μm-thick slices. After dewaxing and antigen repairing, the sections were incubated with antibodies. The primary antibodies used were as follows: Ki67 antibody from Abcam (1:300, Ab15580, USA), EGFR antibody from Proteintech (1:200, 66455-1-lg, China), and CD31 antibody from Proteintech (1:1000, 11265-1-AP, China). The secondary antibodies and DAB solution were obtained from Boster (SV0004, Boster, China). The secondary antibodies for immunofluorescence (IF) staining were goat-anti-rabbit (1:400, ab150080, USA), goat-anti-rabbit (1:400, ab150077, USA) and goat-anti-mouse (1:400, ab150113, USA) antibodies, both obtained from Abcam.

### Cell cycle test

The effect of JorA on the cell cycle was detected by flow cytometry. The cells were fixed in cold 70% ethanol and stained with staining solution (the PI concentration was 1 μg/mL). The stained cells were detected by flow cytometry (Cytoflex, Beckman, USA).

### Apoptosis assay

JorA-induced apoptosis was detected by the Annexin V-FITC staining kit (Beyotime, China). After being rinsing with PBS, the cells were stained with Annexin V-FITC/HO33342, according to the manufacturer's instructions. Fluorescence images were obtained under the fluorescent inverted microscope (Nikon, Eclipse Ti-S, Japan).

### Transcriptomic assay

To further study the effect of JorA on bladder cancer cells at the transcriptional level, transcriptome sequencing analysis was performed. The cells were divided into two groups, and each group contained three samples (5 × 10^6^ T24 cells per sample). RNA was extracted with TRIzol (15596026, Ambion, USA). Transcriptomic analysis was completed by Applied Protein Technology (Shanghai, China).

### Western blotting

To verify the high-throughput omics results and verify the JorA interacting proteins, western blotting experiments were performed. The samples were lysed in RIPA lysate (P0013B, Beyotime, China). The antibodies used in this study were Bcl2 antibody obtained from Wanleibio (1:500, WL01556, China), EGFR antibody obtained from Proteintech (1:3000, 66455-1-lg, China), TOP1 antibody obtained from Proteintech (1:600, 20705-1-AP), FASN antibody obtained from Proteintech (1:800, 10624-2-AP, China), GAPDH antibody obtained from Origene (1:2000, TA802519, USA), and β-actin antibody obtained from Servicebio (1:2000, GB11001, China).

### Labeling biotin on to JorA

In order to reveal the JorA interaction proteins, a streptavidin–biotin pulldown assay was performed. First, biotin was coupled to the hydroxyl group of JorA (coupling was completed by Xi'an Ruixi Biological Technology, China). Then, it was confirmed that the biotin-coupled JorA (biotin-JorA) maintained its anticancer activity by using a CCK8 kit.

### Computer simulation screening of JorA potential interacting proteins

The structural formula of JorA was drawn in ChemDraw software, and the potential interacting proteins of JorA were screened by the online software PharmMapper (http://lilab-ecust.cn/pharmmapper/submitfile.html).

### Pulldown experiment

Streptavidin-coated magnetic beads adsorbed to biotin-JorA were utilized to capture the JorA interaction molecules. To improve the confidence of the pulldown results, three groups were designed in this experiment: the control group, the pulldown (in vivo) group, and the pulldown (in vitro) group. In the pulldown (in vivo) group, the cells were co-incubated with biotin-JorA during cell culture. After the cells were lysed, streptavidin-coated magnetic beads were used for the pulldown experiment. In the pulldown (in vitro) group, the cells were first lysed and then incubated with biotin-JorA. Subsequently, streptavidin-coated magnetic beads were used in the pulldown experiment. The pulled-down components were resuspended with SDT buffer and boiled for 10 min before mass spectrometry detection (mass spectrometry detection was conducted by Applied Protein Technology).

### Molecular docking

The JorA interaction proteins in bladder cancer cells were studied by DockThor [11]. The Protein Data Bank codes for FASN and TOP1 are 1XKT and 1CS7, respectively. Three rotatable bonds of JorA were enabled for the following docking step: the grid box was set to 40 × 40 × 40 Å^3^. Soft docking was enabled in consideration of protein flexibility. The protein–ligand docking results were evaluated based on the binding affinity (kcal/mol). All structure figures were constructed by the program PyMOL (Version 2.0, Schrödinger, LLC).

### Statistical analysis

SPSS 17.0 software was used for statistical analysis. Two-tailed Student's t-test was used to compare the data between two groups. Comparisons among more than two groups were conducted by one-way ANOVA followed by Tukey’s post-hoc analysis. When the P value was less than 0.05, the difference was considered to be significant.

## Results

### JorA specifically inhibited muscle-invasive bladder cancer cells

**Fig. 1:**
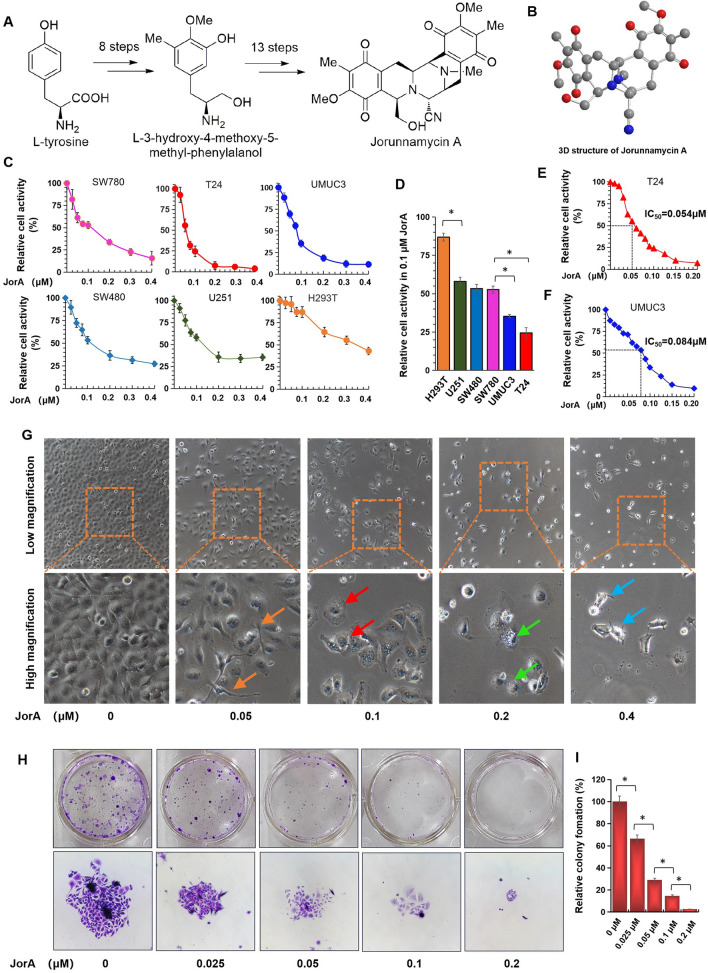
JorA specifically inhibited MIBC cells in vitro*.* **A** Schematic of JorA synthesis steps. **B** The 3D structure of JorA. **C** SW780, T24, UM-UC-3, SW480, U251 and H293T cells were incubated with 0–0.4 μM JorA for 48 h. Cell activity was tested using a CCK-8 kit. **D** SW780, T24, UM-UC-3, SW480, U251 and H293T cells were incubated with 0.1 μM of JorA for 48 h. Data was collected in three independent repeats. *P*-values were derived from Two-tailed Student’s t-test, *p < 0.05. **E** T24 cells were incubated with 0–0.2 μM of JorA. The IC50 is 0.054 μM. **F** UM-UC-3 cells were incubated with 0–0.2 μM of JorA. The IC50 is 0.084 μM. **G** T24 cells were treated by 0–0.4 μM of JorA for 48 h. Cellular morphology changes were examined under a microscope. Orange arrows indicate T24 cells with morphological alteration, red arrows indicate cells with decreased extensibility, green arrows indicate cells with membrane perforation, blue arrow indicated dead cells. **H** T24 cells were incubated with 0–0.2 μM of JorA for 14 days. The cells was fixed by 4% PFA and stained with crystal violet. **I** Quantitative calculation of clonogenic capacity at different concentrations of JorA. The statistical difference was analyzed by one-way ANOVA followed by Tukey post-hoc analysis, *p < 0.05

As shown in Fig. 1A, JorA was synthesized from l-tyrosine according to the methods of the authors’ previous study [10]. Additionally, chemical structures of JorA were further characterized by ^1^H NMR and ^13^C NMR (see Additional file 1: Fig. S1A and S1B). The 3D structure of JorA is shown in Fig. 1B. Human embryo kidney 293 T cells; glioma U251 cells; colorectal cancer SW480 cells; and bladder cancer SW780, UM-UC-3, and T24 cells were treated with 0–0.4 μM of JorA. As shown in Fig. 1C and D, different cell lines have different sensitivities to JorA, and bladder cancer cell lines have higher sensitivity to JorA than normal cells and other cancer cells. In bladder cancer cell lines, MIBC lines T24 and UM-UC-3 were more sensitive to JorA than NMIBC cell line SW780, suggesting that JorA may selectively inhibit MIBC. The IC_50_ of T24 and UM-UC-3 cells was 0.054 and 0.084 μM, respectively (Fig. 1E and F). Morphological results (Fig. 1G) showed that 0.05 μM JorA elongated and narrowed T24 cells, 0.1 μM JorA caused cells to lose extensibility, 0.2 μM JorA resulted in cell membrane perforation, and 0.4 μM JorA induced detaching. Moreover, the inhibition of JorA on T24 was also studied through a colony formation experiment. As shown in Fig. 1H and I, JorA inhibited the cloning of T24 in a concentration-dependent manner. The effect of JorA on morphology and clonal formation of bladder cancer UM-UC-3 cells is shown in Additional file 2: Fig. S2.

### JorA represses xenografted bladder tumor growth in vivo

**Fig. 2:**
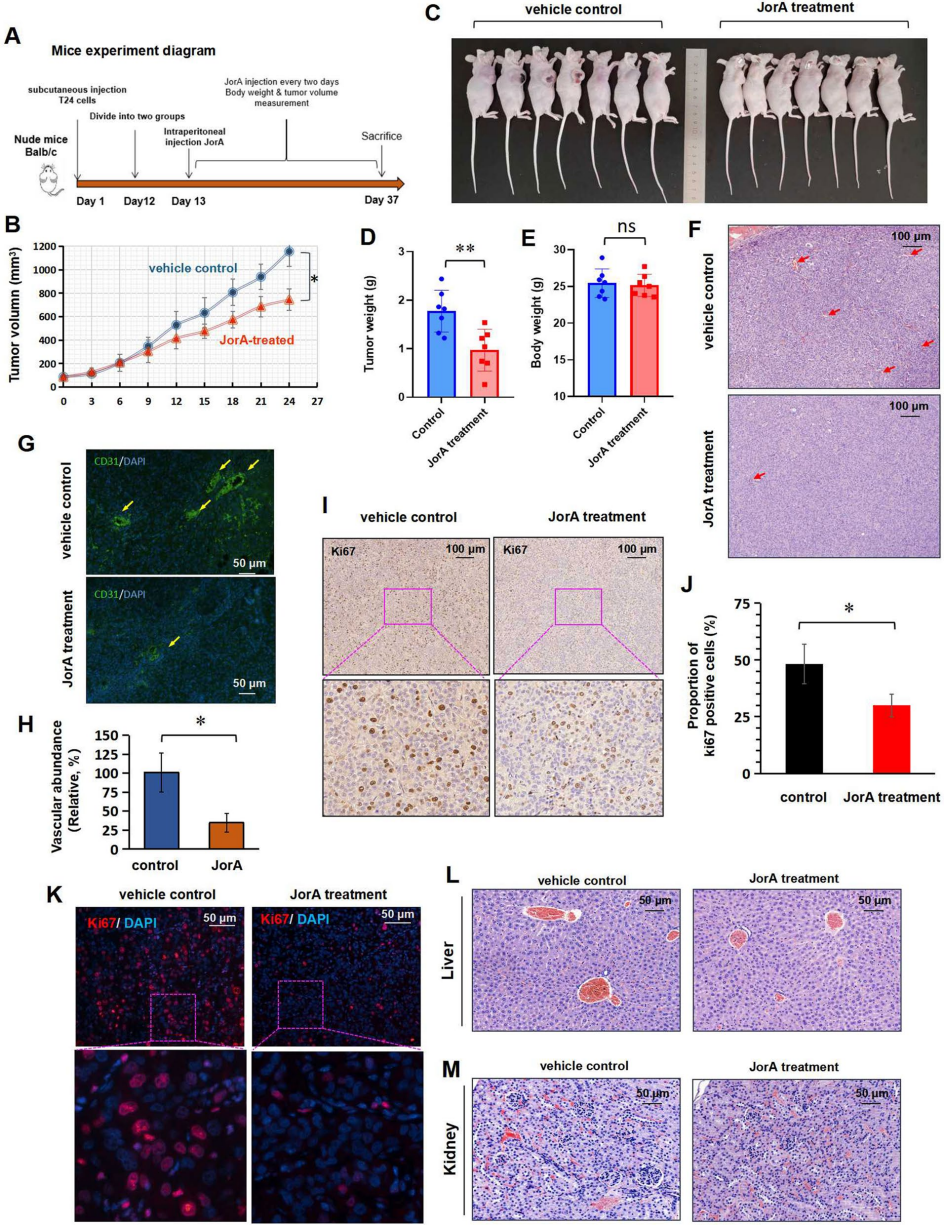
JorA represses xenografted bladder tumors growth in vivo*.* **A** Diagram of xenograft experiment. **B** Curves of the xenografted tumor volume in two groups of mice. The statistical difference was analyzed by one-way ANOVA, *p < 0.05. **C** The photos of mice body (with subcutaneous tumors). **D** Tumor weight of mice in both groups. P-values were derived from Two-tailed Student’s t-test, **p < 0.01. **E** Body weight of mice in both groups. **F** H&E staining photos of xenografted tumor sections. The arrows indicate the vascular section in the slice. **G** IF staining for vascular marker CD31 xenografted tumor sections. The arrows indicate the vascular section in the slice. **H** Quantitative contrast map of abundant blood vessels in xenografted tumor. The ordinate represents the relative abundance of blood vessels (take control group as 100%). P-values were derived from Two-tailed Student’s t-test, *p < 0.05. **I** Immunohistochemical staining results of Ki67 in xenografted tumor sections. **J** Quantitative contrast map of Ki67. The ordinate represents the proportion of Ki67 positive cells. P-values were derived from Two-tailed Student’s t-test, *p < 0.05. **K** IF staining results of Ki67 in xenografted tumor sections. **L** H&E staining photos of mice liver sections. **M** H&E staining photos of mice kidney sections

To investigate the anticancer activity of JorA on bladder tumors in vivo, a xenograft experiment was performed using a mouse model (Fig. 2A). By measuring the changes in tumor volume, it was found that JorA could slow the growth rate of subcutaneous tumors in mice (Fig. 2B). The xenografted tumors are shown in Fig. 2C, and the weights of tumors and mouse bodies were quantitatively and statistically compared. As shown in Fig. 2D and E, JorA significantly reduced tumor weight but did not significantly affect mouse body weight. H&E-staining and CD31 IF-staining results indicated that the abundance of blood vessels of tumors in the JorA treatment group was significantly lower than that of the control group (Fig. 2F–H). The results of IHC and IF showed that JorA significantly reduced the proportion of Ki67-positive cells of bladder tumors (Fig. 2I–K). These results demonstrated that JorA effectively inhibited bladder tumors in vivo. H&E staining of liver and kidney slices showed that JorA had no significant effect on the liver and kidney tissues of mice (Fig. 2L and M).

### JorA modulated cell proliferation- and apoptosis-related molecules in MIBC T24 cells

**Fig. 3:**
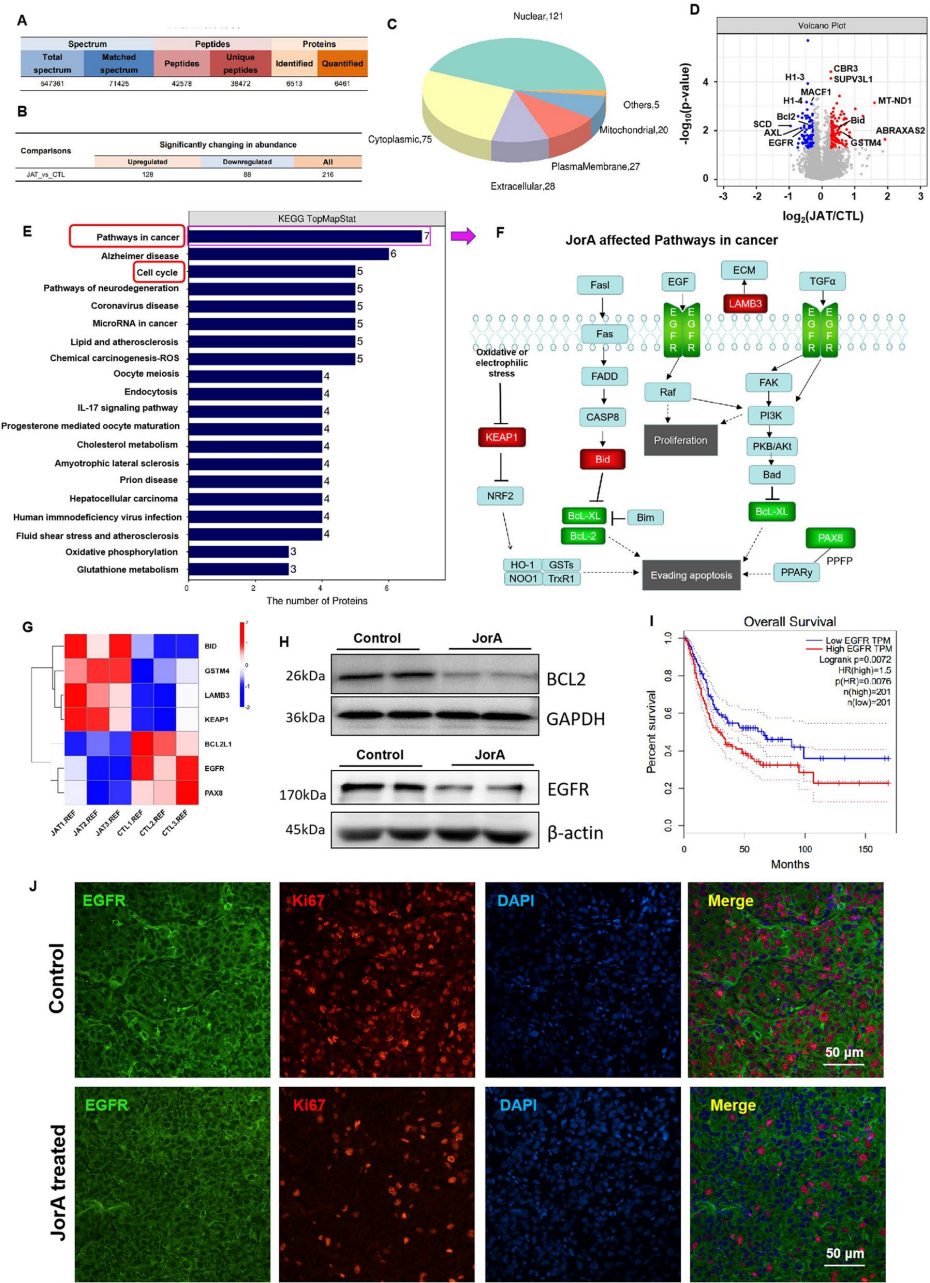
Proteomics analysis reveals JorA modulates proliferation and apoptosis related molecules in MIBC T24 cells. **A** The identification and quantitative results statistics of spectrum, peptides and proteins. **B** The number of significantly upregulated and downregulated proteins by JorA. **C** Pie chart of proteomics organelle localization analysis. **D** Volcanic map of JorA induced significantly changed proteins. Gray dots indicate genes with no significant difference, red dots indicate upregulated genes with significant differences, and blue dots indicate downregulated genes with significant differences. **E** Top 20 pathways of KEGG enrichment analysis result. **F** JorA modulated key molecules (such as KEAP1, Bid, LAMB3, EGFR, Bcl-XL, Bcl-2, and PAX8) in “pathways in cancer”. Red boxes indicate upregulated molecules, and green boxes indicate downregulated molecules. **G** Heat map of JorA-modulated molecules in "paths in cancer". CTL refers to the control group; JAT refers to the JorA treatment group. **H** Western blotting to test the protein level of Bcl-2 and EGFR. **I** The relationship between EGFR expression level and survival rate of bladder cancer patients. **J** The expression levels of Ki67 and EGFR in xenografted bladder tumor were detected by IF staining

Proteomics analysis identified 6,513 proteins, of which 6,461 were quantified in this study (Fig. 3A). With JorA treatment, 216 significantly changed proteins were found, of which 128 were upregulated and 88 were downregulated by JorA (Fig. 3B). Organelle localization analysis showed that JorA-affected molecules majorly localized to the nucleus, followed by to the cytoplasm, extracellular area, plasma membrane, mitochondria, and other regions (Fig. 3C). The volcano plot showed that the protein expression of Bid, Bcl-2, EGFR, and SCD, among other genes, was significantly affected by JorA (Fig. 3D). KEGG enrichment analysis showed that “pathways in cancer” ranked first (Fig. 3E). Specifically, the molecules involved in cancer-related pathways, including LAMB3, KEAP1, Bid, EGFR, Bcl-XL, Bcl-2, and PAX8, were all regulated by JorA (Fig. 3F). The proteomic heatmap quantitatively showed that JorA upregulated LAMB3, KEAP1, and Bid gene expression, and downregulated BCL2L1, EGFR, and PAX8 gene expression (Fig. 3G). Moreover, the “EGFR tyrosine kinase inhibitor resistance” pathway was also enriched by KEGG enrichment analysis (Additional file 3: Fig. S3). JorA modulating EGFR and BCL2 was also demonstrated by western blotting (Fig. 3H). By analyzing the survival rate of EGFR in patients with bladder cancer through an online clinical database (http://gepia.cancer-pku.cn/), it was found that the survival rate of the high EGFR expression group was significantly lower than that of the low EGFR expression group, suggesting that high EGFR expression may be a sign of malignant transformation of bladder tumors. Inhibition of EGFR expression by JorA would be beneficial to improve the survival of patients with bladder cancer (Fig. 3I). The inhibition of JorA on EGFR and Ki67 was also verified in the xenografted tumor section of the mouse model (Fig. 3J).

### Phenotype evidence of JorA repressing proliferation and migration and inducing apoptosis

**Fig. 4:**
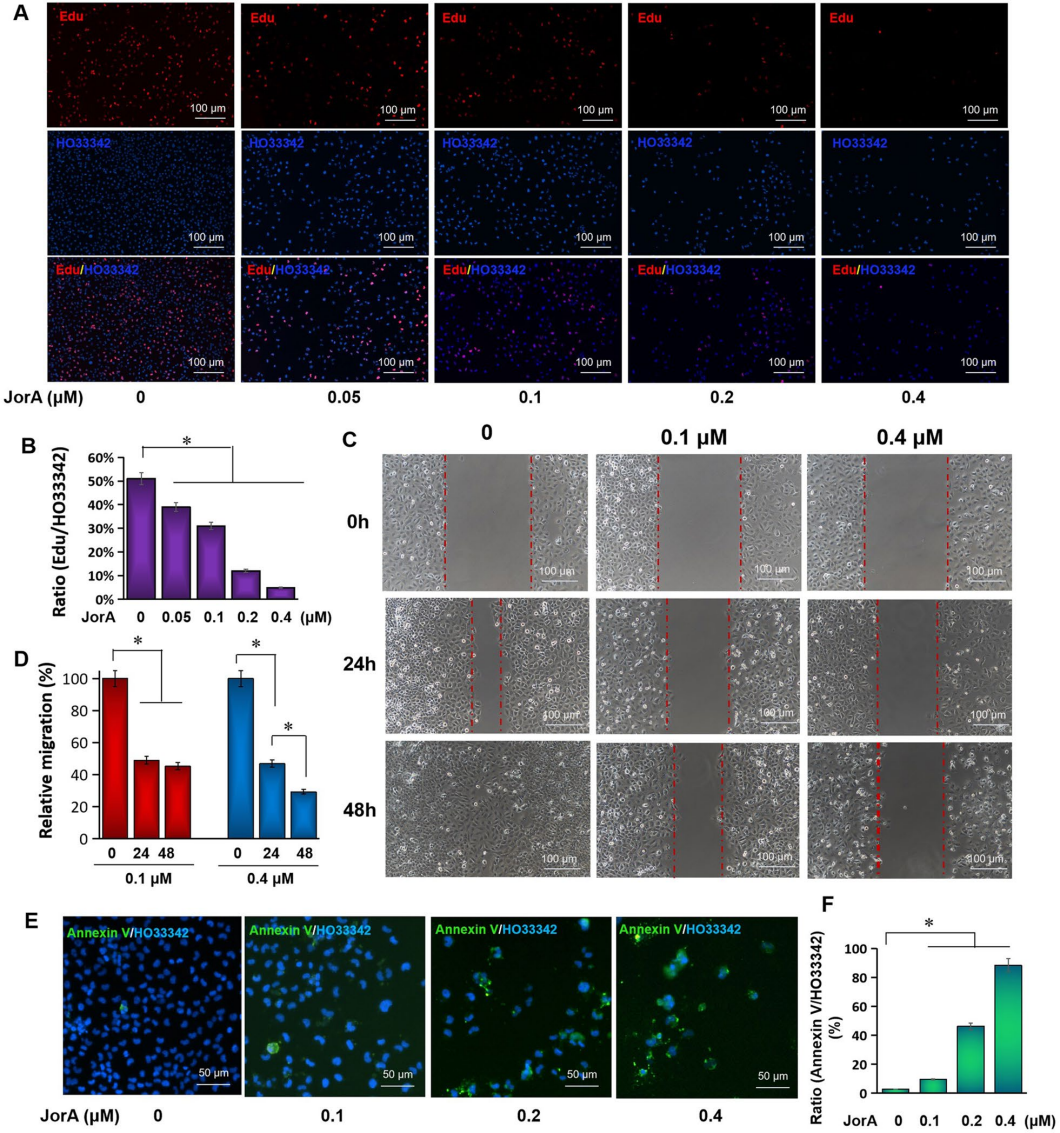
Phenotype evidence of JorA repressing proliferation, migration and inducing apoptosis. **A** EdU staining to investigate JorA inhibiting DNA replication in bladder cancer T24 cells. EdU positive stained cells were shown as red fluorescence; Cells nuclei were shown as blue fluorescence. **B** Quantitative analysis chart of EdU experiment results. The statistical difference was analyzed by one-way ANOVA followed by Tukey post-hoc analysis, *p < 0.05. **C** The scratch test was employed to observe cell migration. **D** Quantitative analysis of cell migration experiment results. The relative migration rate of control group (without JorA) of 48 h was 100%. The statistical difference was analyzed by one-way ANOVA followed by Tukey post-hoc analysis, *p < 0.05. **E** To test apoptosis, Annexin V-FITC/HO33342 staining was performed. Annexin V-FITC positive cells were stained as green fluorescence, and the cells nuclei were stained as blue fluorescence. **F** Quantitative analysis of JorA-induced apoptosis. The statistical difference was analyzed by one-way ANOVA followed by Tukey post-hoc analysis, *p < 0.05

Since the above TMT proteomics data show that JorA repressed MIBC cells through inhibiting proliferation, dampening migration, and inducing apoptosis, experiments were performed to verify these phenotypes. As shown in Fig. 4A, the DNA replication rate was quantified by the ratio of EdU/HO33342. It showed that, as the concentration of JorA increased, the DNA replication speed decreased, which indicated that JorA attenuated proliferation by suppressing DNA replication in a dose-dependent manner (Fig. 4B). Figure 4C shows that the rate of scratch healing of MIBC T24 cells was obviously suppressed by JorA. Moreover, the quantitative calculation results showed that JorA significantly mitigated the migration of T24 cells (Fig. 4D). To detect the JorA-induced apoptosis, phosphatidylserine was stained with ANNIX-V-FITC fluorescent dyes. Figure 4E and F show that JorA significantly promoted apoptosis in a dose-dependent manner, which was consistent with the proteomic results.

### The effect of JorA on MIBC transcription level

**Fig. 5:**
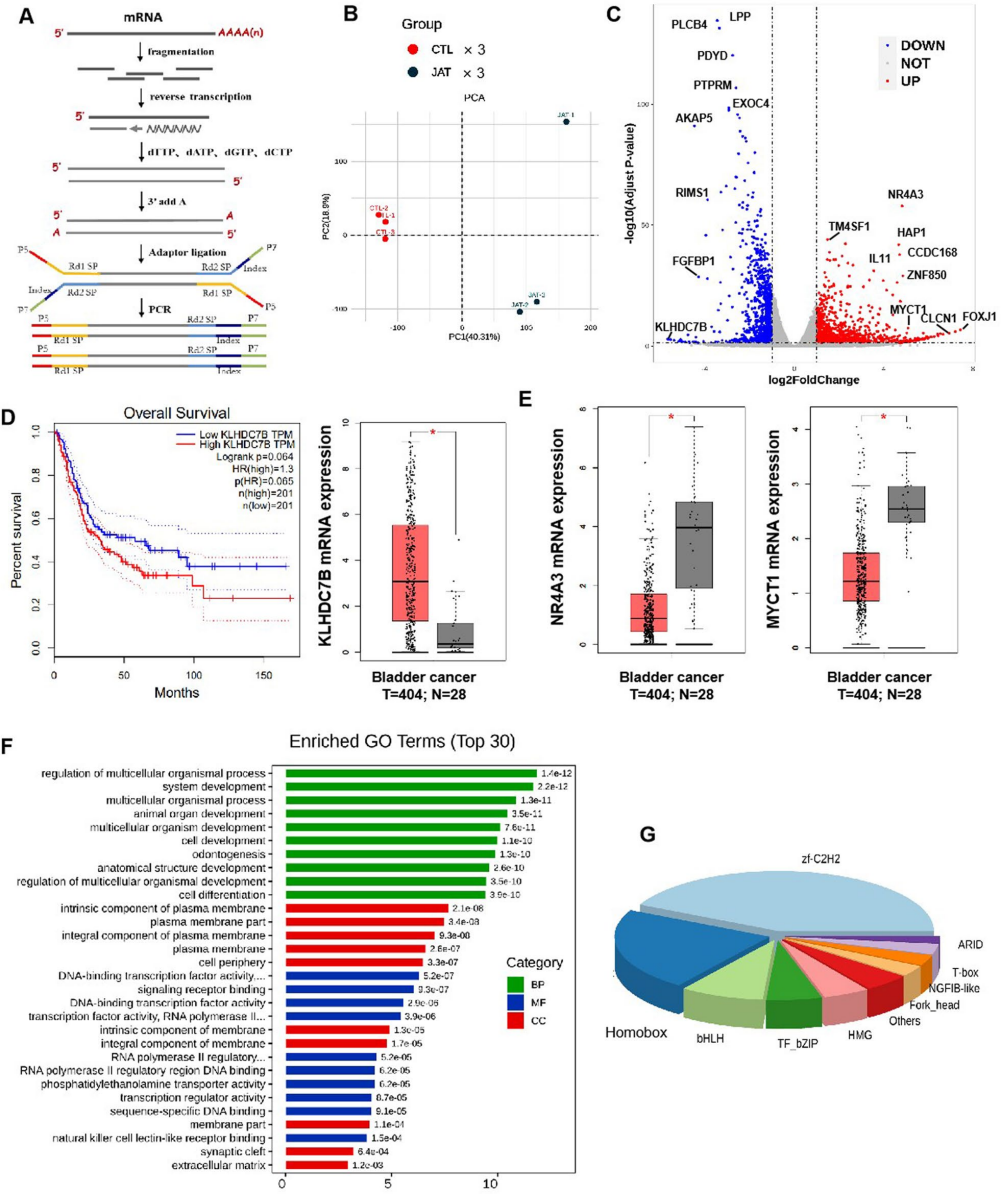
The effects of JorA on the transcription level of MIBC cells. **A** The diagram of transcriptomics library construction. **B** Principal component analysis of control group (CTL) and JorA treatment group (JAT). **C** Volcanic map of transcripts whose expression levels are significantly affected by JorA. Gray dots refer to genes with no significant difference; red dots refer to upregulated genes with significant differences, and blue dots refer to downregulated genes with significant differences. **D** Left: The relationship between the KLHDC7B expression level and the survival rate of bladder cancer patients; Right: The differential expression of KLHDC7B in bladder tumor tissues and normal bladder tissues. Data are obtained from GEPIA (http://gepia.cancer-pku.cn/). **E** The differential expression of RNF116 and NKD1 in bladder tumor tissues and normal bladder tissues. Data are obtained from GEPIA. **F** Top 30 pathways in GO enrichment analysis of JorA-induced significantly changed genes. **G** The transcription factors of gene with significant changes caused by JorA are displayed in pie chart

To investigate the potential pharmacological mechanism of JorA inhibiting MIBC T24 cells, transcriptomic analysis was performed. Transcriptome library construction and sequencing procedures are shown in Fig. 5A. Principal component analysis of the control groups (CTL) and JorA treatment group (JAT) showed good results (Fig. 5B). Volcano plots of transcriptomic results showed that JorA altered the mRNA level of FOXJ1, KLHDC7B, PLCB4, CLCN1, and CCDC168, among other genes in T24 cells (Fig. 5C). It was found that KLHDC7B, the mRNA downregulated the most by JorA, was highly expressed in bladder cancer and associated with a poor prognosis in patients with bladder cancer (Fig. 5D). NR4A3 and MYCT1 genes, whose expression was increased by JorA, were found to be lowly expressed in bladder cancer samples in clinical cases (Fig. 5E). Transcriptomic Gene Ontology (GO) enrichment analysis results showed that the dominant impact of JorA on MIBC T24 cells mainly focused on biological process (BP)-related pathways (Fig. 5F). By using enrichment analysis for transcription factors of JorA-induced significantly altered mRNA, it was discovered that transcription factor zf-C2H2 ranked first, followed by Homobox, indicating that JorA might affect these transcription factors (Fig. 5G). Homobox has been reported as a cancer treatment target in pancreatic cancer [12].

### Combined analysis of transcriptomic and proteomic results

**Fig. 6:**
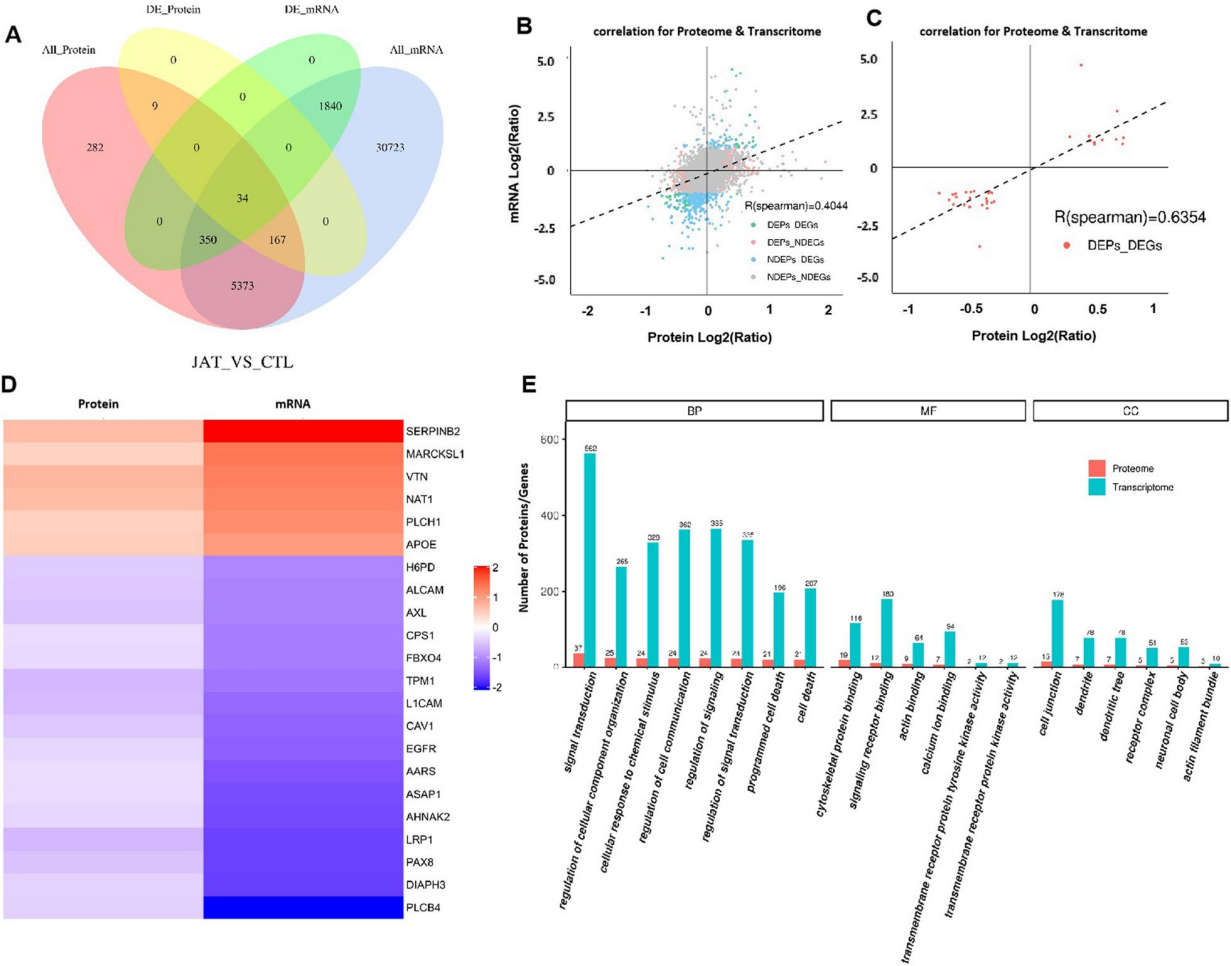
The combined analysis of proteome and transcriptome. **A** The Venn diagram of proteomic and transcriptomic result. All_Protein represents all the quantifiable proteins of the proteome; All_mRNA represents all the quantifiable genes obtained from the transcriptome; DE_Protein represents the differentially expressed proteins; DE_mRNA represents the differentially expressed genes. **B** Overall analysis of correlation between proteome and transcriptome. NDEPs means no differentially expressed protein; NDEGs means no differentially expressed gene (mRNA); DEPs means differentially expressed proteins; DEGs means differentially expressed genes (mRNA). **C** Correlation diagram of differentially expressed proteins and differentially expressed genes (mRNA). Red dots (DEPs_DEGs) represent differentially expressed proteins and genes. **D** A heatmap for clustering analysis of differentially expressed proteins and genes (mRNA). **E** GO enrichment of combined analysis between proteomics and transcriptomics results. Red column represents proteome; cyan column represents transcriptome

To further explore the molecular network mechanism of JorA against MIBC cells, combined analysis of transcriptomics and proteomics was performed. A Venn diagram of conjoint analysis is shown in Fig. 6A. Overall, 34 genes were differentially expressed at the levels of both mRNA and protein in the JorA treatment group. Combined analysis of all quantified proteins and their associated mRNAs showed that the correlation coefficient was 0.4044, indicating their positive correlation (Fig. 6B). The correlation between differential proteins and differential mRNAs showed that the correlation coefficient was 0.6354, indicating a better positive correlation (Fig. 6C). Cluster analysis of differential proteins and related differential mRNAs revealed that 22 molecules (including EGFR) were affected by JorA at both the protein level and the transcription level in a consistent trend (Fig. 6D). In addition, the cellular molecule pathways involving EGFR were most enriched, suggesting that EGFR might be the essential molecule of JorA suppressing MIBC (Additional file 4: Fig. S4). As shown in Fig. 6E, the GO enrichment of conjoint double omics analysis showed that the main impact of JorA against MIBC T24 cells was clustering in the BP pathways, which is consistent with what is shown in Fig. 5F.

### Exploration of molecules interacting with JorA in MIBC type cells

**Fig. 7:**
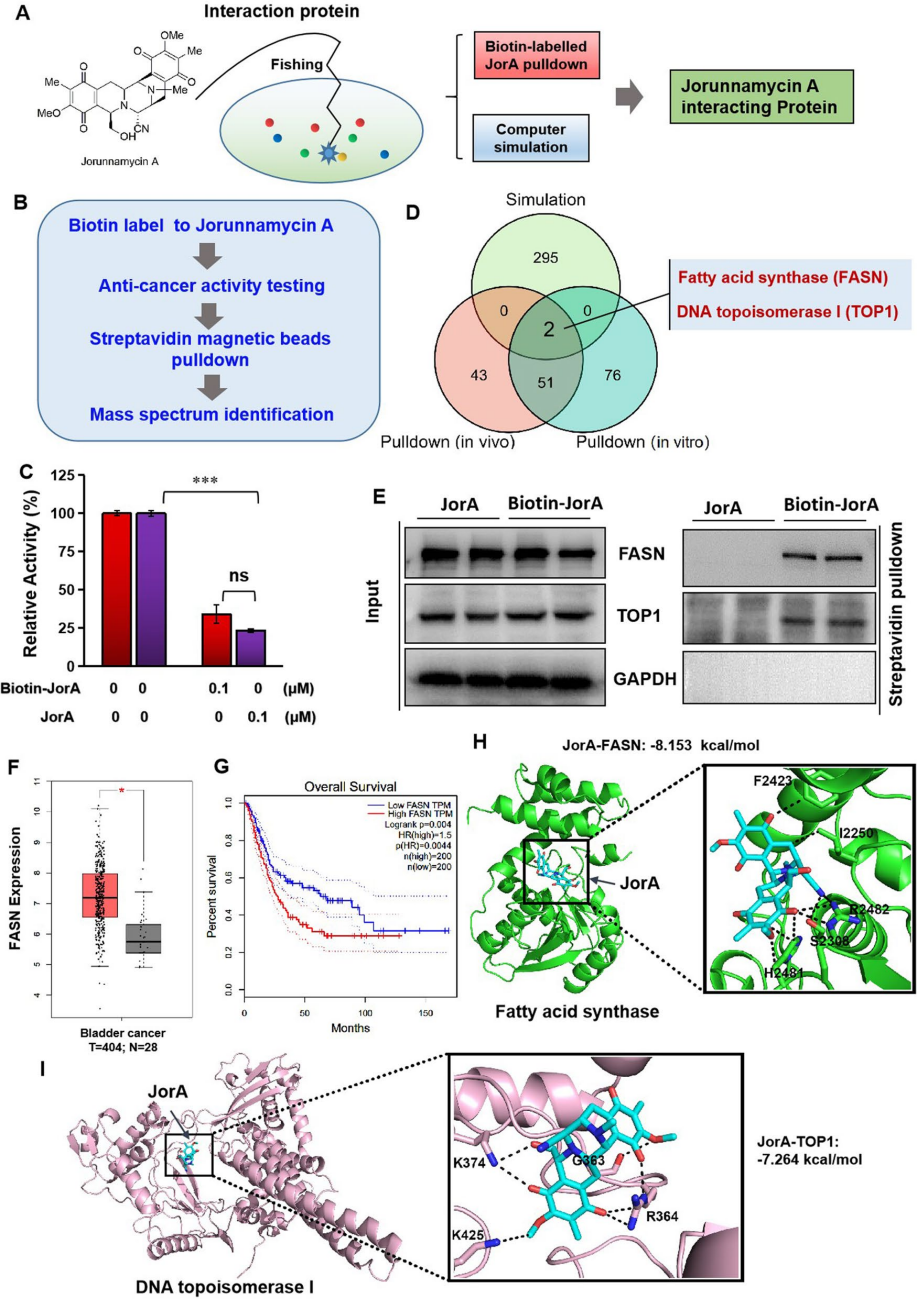
Studies on JorA-interaction proteins. **A** Schematic diagram of fishing JorA-interaction proteins. **B** The protocol for biotin-JorA pulling-down experiment. **C** Comparison of the anticancer activity between biotin-JorA and native JorA. Data was collected in three independent repeats. Error bars represent standard deviation. *P*-values were derived from Two-tailed Student’s t-test; ***p < 0.001; ns means no significant. **D** The Venn diagram of three mode samples mass spectrum identification results is shown in the figure. Simulation represents computeral simulation through Pharm Mapper platform (http://lilab-ecust.cn/pharmmapper/), pulldown (in vivo) and pulldown (in vitro) has been explained in Materials and Methods section. **E** Pulldown followed by western blotting to test the JorA-interaction proteins FASN and TOP1. **F** The differential expression of FASN in bladder tumor tissues and normal bladder tissues. Data are obtained from GEPIA. **G** The relationship between the FASN expression level and the survival rate of bladder cancer patients. **H** The docking structure of molecular interaction between JorA and FASN. JorA and FASN were colored cyan and green respectively. **I** The docking structure of molecular interaction between JorA and TOP1. JorA and TOP1 were colored cyan and lightpink respectively

To further explore the pharmacological molecular mechanism, the JorA interaction molecules in MIBC cells were investigated. Computer simulation and biotin-JorA pulldown assays were performed to screen the molecules interacting with JorA (Fig. 7A). Before the pulldown experiment, biotin was labeled on to the hydroxyl site of JorA and the cytotoxicity of biotin-labeled JorA (biotin-JorA) was tested against T24 cells. The purity of biotin-JorA was tested by HPLC (Additional file 5: Fig. S5A). The ^1^H NMR and MS spectra of biotin-JorA are shown in Additional file 5: Fig. S5B and S5C, respectively. The pulldown experiment procedure diagram is shown in Fig. 7B. As shown in Fig. 7C, biotin-JorA had equal antiproliferation activity to native JorA. As shown in Fig. 7D, the intersection of the computer simulation and pulldown results contained two molecules: FASN and TOP1. To verify the result, pulldown assay followed by western blotting were performed. As shown in Fig. 7E, biotin-JorA was able to interact with FASN and TOP1 (as a control, GAPDH could not bind with biotin-JorA), which is consistent with what is shown in Fig. 6D. From the clinical data, the expression level of FASN in bladder tumor tissues was significantly higher than that in normal tissues (Fig. 7F), and in patients with bladder cancer, the survival rate of the high expression group of FASN was significantly lower than that of the low expression group (Fig. 7G). The clinical database suggested that higher FASN was associated with a poor prognosis in patients with bladder cancer, indicating that FASN might be the bladder cancer therapy target. It was found that JorA bound to the thioesterase domain of FASN with a binding affinity of − 8.153 kcal/mol (Fig. 7H). The thioesterase activity of FASN was demonstrated to be positively related to carcinogenesis [13]. The JorA-binding amino acids, serine 2,308 and histidine 2,481, are located in the thioesterase active center of FASN, suggesting that JorA inhibits the thioesterase activity of FASN. TOP1 is a key enzyme in the process of DNA replication, and the reduction of TOP1 activity can directly lead to cell cycle arrest. The binding affinity was − 7.264 kcal/mol and the binding sites are shown in Fig. 7I.

## Discussion

**Fig. 8:**
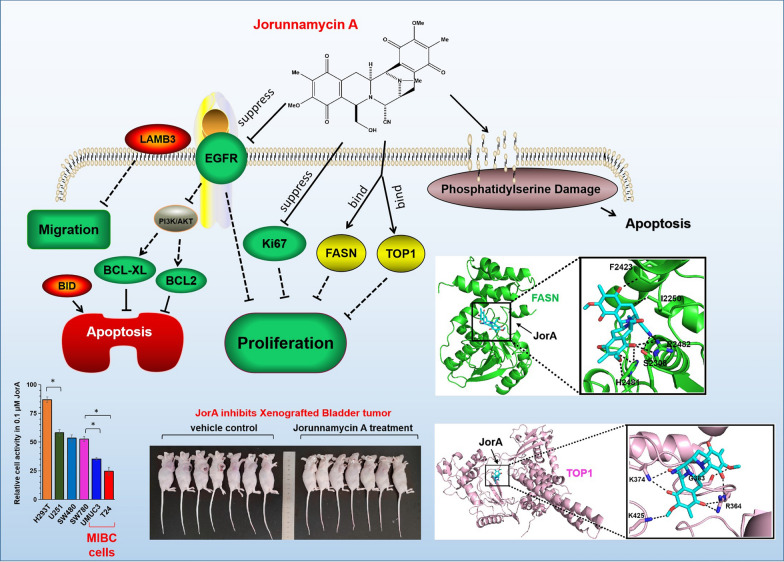
The graphic abstract of JorA inhibiting MIBC. JorA plays anticancer roles mainly through repressing the proliferation and migration, and inducing apoptosis of MIBC. The green oval labeled molecules are those significantly downregulated by JorA, and the red ovals labeled molecules are those significantly upregulated by JorA. The molecules marked with yellow oval are the interacting molecules of JorA. In the insert, the anticancer activity of JorA on MIBC and the 3D structures of the molecular interaction of JorA were displayed

Because different patients with bladder cancer have different needs, bladder cancer therapy approaches are complicated [14]. The ideal anticancer drug should have obvious inhibitive or even killing effects on cancer cells, but no or few effects on normal cells. In this study, the special inhibitory effect of JorA against MIBC was identified for the first time: JorA has special cytotoxicity to MIBC and low cytotoxicity to normal somatic cells. It was discovered that JorA repressed proliferation and migration and induced apoptosis in MIBC cells. The potential mechanism of JorA against bladder cancer cells was revealed based on proteomic and transcriptomic investigation (Fig. 8).

EGFR-related signaling pathways are the main targeted pathways of JorA (Additional file 3: Fig. S3). JorA significantly decreased EGFR at both mRNA and protein levels. EGFR has been a topic of focus in research because of its importance in tumorigenesis [15]. EGFR gene mutation and amplification have been shown to be crucial drivers of many cancer types [16]. The role of EGFR in non-small cell lung cancer, glioblastoma, and basal-like breast cancer has inspired much research and drug development [17, 18]. Tyrosine kinase inhibitors (most notably gefitinib and erlotinib) have excellent therapeutic effects on EGFR mutated/amplified tumors [19]. However, mutations in EGFR have been found to cause cancer cells to be resistant to these drugs [20]. In this study, JorA was found to reduce EGFR-involved tyrosine kinase inhibitor drug resistance via downregulated EGFR, AXL, and Bcl2 family members (Additional file 3: Fig. S3). In bladder cancer T24 cells, AXL was significantly downregulated by JorA. AXL is a member of the Tyro3-Axl-Mer receptor tyrosine kinase subfamily [21]. It has been reported to confer intrinsic osimertinib resistance and promote the emergence of tolerant cells [22]. AXL is also known to maintain cell survival and enhance cell tolerance to osimertinib through association with EGFR [22]. Since the EGFR/AXL pathway plays a critical role in the emergence of osimertinib resistance, JorA might be beneficial for inhibiting osimertinib resistance by downregulating EGFR and AXL.

JorA targets "pathways in cancer" to play anticancer roles by enhancing apoptosis in MIBC cells. Here, it was found that Bcl2, Bcl-XL, Bid, and Pax8, which are closely related to apoptosis, can be regulated by JorA. It has also been reported that the overexpression of Bcl-2 is associated with drug resistance and a poor clinical outcome in patients with various cancer types, and Bcl-2 has been considered as a potential cancer therapy target [23]. Bcl-xL is a 30 kDa anti-apoptotic protein belonging to the Bcl-2 family that can inhibit apoptosis through two different mechanisms: (1) suppressing apoptosis by heterodimerization with apoptotic proteins; (2) maintaining the normal membrane state under stress by direct pore formation on the outer membrane of mitochondria [24]. Bid protein is a pro-apoptotic protein in the Bcl-2 family. It has the function of highly inducing cytochrome c leakage from mitochondria to the cytoplasm by caspase 8 digestion, thus, plays an important positive regulatory role in cell apoptosis [25]. PAX8, a transcription factor belonging to the PAX gene family, has been observed to be expressed in tumorigenesis and has been demonstrated to facilitate malignant development. It has been widely considered as a potential new target for malignant tumor treatment [26, 27]. The discovery in the present study that JorA increases Bid expression and decreases Bcl2, Bcl-XL, and Pax8 expression suggests that JorA accelerates the apoptosis of bladder cancer cells from multiple angles.

JorA impacts transcription factors in MIBC cells. The enrichment analysis of transcription factors to the transcriptome showed that transcription factor zinc finger-C2H2 (zf-C2H2) occupied the primary position. zf-C2H2 has the functions of recognizing and binding specific DNA fragments and participating in the regulation of gene expression, playing essential roles in various biological processes, including early embryogenesis, development, cell differentiation, cell proliferation, and cell death [28, 29]. Researchers have found that zf-C2H2 can not only bind DNA, but also partner with a variety of proteins to regulate gene expression. Moreover, it can not only suppress genes, but also start genes, and even assist in DNA packaging [30]. However, the understanding of C2H2 transcription factors is currently insufficient, and whether zf-C2H2 can be a tumor therapy target remains to be further studied.

JorA significantly affects the transcriptome of MIBC cells, and these transcripts whose expression levels are significantly altered by JorA have been reported to be closely related to bladder cancer. KLHDC7B is the most downregulated transcript among all transcripts with significant changes caused by JorA (Fig. 4C). The clinical database showed that KLHDC7B was highly expressed in patients with bladder tumors, and the survival rate of the high expression group in patients with bladder tumors was significantly lower than that of the low expression group (Fig. 4D), suggesting that the high expression of KLHDC7B may be one of the reasons for bladder tumor occurrence and a poor prognosis. KLHDC7B has been demonstrated to be a marker in urine exosomes that can be used to detect the tumor progression of bladder cancer, and its mRNA imbalance is related to tumor stage and grade as well as hematuria degree [31]. MYCT1, a gene encoding the tumor suppressor protein myc target 1, was discovered to be upregulated by JorA. MYCT1 has been reported as a novel tumor suppressor gene, whose target gene is the famous oncogene myc [32]. Decreased expression of MYCT1 has been considered to be positively related to carcinogenesis [33]. Moreover, MYCT1 has also been reported to inhibit EMT and the migration of laryngeal cancer cells via the SP1/miR-629-3p/ESRP2 pathway [34]. These factors suggest that JorA upregulating MYCT1 has a positive effect on the suppression of bladder cancer. NR4A3 is a gene coding the nuclear receptor subfamily 4 group A member 3, which belongs to a steroid-thyroid hormone-retinoid receptor superfamily [35]. NR4A3 was identified as a tumor suppressor for acute myeloid leukemia (AML): deletion of NR4A3 led to the rapid development of AML in mice, and NR4A3 gene deletion is common in patients with AML [36]. In a bladder cancer model, it was found that silencing NR4A3 in bladder cancer T24 and 5637 cells could enhance cell proliferation and migration, whereas overexpression of NR4A3 could inhibit the malignant phenotype of bladder cancer [37]. Here, it was found that NR4A3 was significantly lower expressed in patients with bladder cancer, whereas JorA could increase NR4A3 expression in bladder cancer T24 cells, suggesting that JorA may exert its tumor suppressor effect through the regulation of NR4A3.

A highlight of this study is that the anticancer mechanism was not only revealed in a panoramic perspective but also elaborated the interaction targets of JorA in a micro-perspective. In particular, to study the JorA interaction protein in bladder cancer cells, two approaches were used (computer simulation and biotin-streptavidin pulldown assays). Finally, all results of the above models point to FASN and TOP1 being the interacting proteins of JorA. Notably, FASN and TOP1 are known as tumor promoting factors and biomarkers of a poor prognosis. TOP1 encodes DNA that resolves topological constraints and plays an important role in DNA replication and gene transcription [38]. It has been reported that TOP1 inhibitors can provide strong tools to restrain DNA replication and could be helpful for establishing new strategies for efficient cancer treatments [39]. So far, there are FDA-approved drugs available, such as Topotecan and Irinotecan [40]. FASN is a lipogenic enzyme that is considered as a metabolic reprogramming cancer hallmark. Upregulation of FASN has been found to accompany many human cancers’ natural history and is related to a poor prognosis, including bladder cancer, colorectal cancer, and pancreatic cancer [41]. To support rapid proliferation, tumor cells utilize fatty acids as an energy source more than normal cells. Therefore, key enzymes involved in lipid metabolism pathways such as FASN are closely associated with the initiation and progression of tumors [41]. Mutant oncogenic RAS gene is an critical driving force for the progression of bladder tumors [42]. Mutated RAS can drive the expression of adipogenic genes such as FASN to activate the lipid synthesis pathway [43]. Our study suggests that the inhibition of FASN by JorA may provide a basis for therapeutic strategies targeting bladder cancer lipid metabolism. Since FASN has been demonstrated to intertwine with various signaling networks to initiate carcinogenesis and accelerate cancer progression, FASN has been widely considered as a tumor therapeutic target [44]. JorA is able to bind to the active center of FASN and inhibit its thioesterase activity, thus, suppressing the promotion of FASN on tumors.

## Conclusions

In this study, it was discovered that a marine-derived compound, JorA, effectively represses MIBC via inhibiting proliferation and inducing apoptosis, mainly through an EGFR-involved pathway. The pharmacological mechanism was also revealed: in the perspective of proteomics, JorA mainly modulates the expression level of EGFR, Bid, and Bcl, which belong to the "pathways in cancer" signaling pathway. At the microscopic level of molecular interactions, JorA combines with two important tumor-promoting molecules, FASN and TOP1, and inhibits tumor progression. The findings of this study suggest that JorA has potential to be developed into a promising bladder cancer therapeutic drug.

### Supplementary Information

**Additional file 1: Fig. S1.** NMR spectrum of JorA. (A) ^1^H NMR spectrum of JorA (400 MHz, CDCl_3_). (B) ^13^C NMR spectrum of JorA (100 MHz, CDCl_3_).

**Additional file 2: Fig. S2.** JorA inhibited bladder cancer UM-UC-3 cells in vitro. (A) UM-UC-3 cells were treated by 0 − 0.4 μM of JorA for 48 h. Cellular morphology changes were examined under a microscope. Red arrows indicate the UM-UC-3 cells with decreased extensibility, green arrows indicate cells with membrane perforation, blue arrow indicated dead cells. (B) UM-UC-3 cells were incubated with 0 − 0.4 μM of JorA for 14 days. The cells was fixed by 4% PFA and stained with crystal violet.

**Additional file 3: Fig. S3.** KEGG map of JorA down-regulating the key molecules of EGFR tyrosine kinase inhibitor resistance pathway. The KEGG enrichment result of differential proteome indicated that JorA could affect EGFR tyrosine kinase inhibitor resistance pathway by inhibiting EGFR, AXL and Bcl-2. The molecules in the bright green square represent the molecules that are significantly downregulated by JorA, and the fold changes are shown in yellow text.

**Additional file 4: Fig. S4.** JorA mainly targeted EGFR involved pathways. Combined analysis of transcriptome and proteome showed that the EGFR related pathway was dominant in the JorA-induced transcriptome and proteome alteration.

**Additional file 5: Fig. S5.** Identification of biotin-labeled JorA. (A) HPLC data of biotin-labeled JorA. (B) ^1^H NMR spectrum of biotin-labeled JorA (400 MHz, CDCl_3_). (C) MS spectrum of biotin-labeled JorA.

## Data Availability

The transcriptome data are deposited to NCBI Sequence Read Archive and available at https://submit.ncbi.nlm.nih.gov/subs/sra/SUB12153669/overview (BioProject ID: PRJNA891952). The mass spectrometry proteomics data have been deposited to the ProteomeXchange Consortium (http://proteomecentral.proteomexchange.org) via the iProX partner repository with the dataset identifier PXD037638 (http://proteomecentral.proteomexchange.org/cgi/GetDataset?ID=PXD037638). The other data are available in the main text or the additional file.

## Abbreviations

CCK8: Cell Counting Kit-8
EdU: 5-Ethynyl-2′-deoxyuridine
EGFR: Epidermal growth factor receptor
FASN: Fatty Acid Synthase
IHC: Immunohistochemistry
JorA: Jorunnamycin A
KLHDC7B: Kelch Domain Containing 7B
MIBC: Muscle invasive bladder cancer
NR4R3: Nuclear receptor subfamily 4 group A member 3
PDB: Protein data base
TMT: Tandem mass tags
TOP1: Topoisomerase1
