# Supplementary figures and images for: Integrated multi-omics analyses reveal Jorunnamycin A as a novel suppressor for muscle-invasive bladder cancer by targeting FASN and TOP1
Source: J Transl Med. 2023 Aug 16;21:549. doi: 10.1186/s12967-023-04400-3 (PMC10428641; doi:10.1186/s12967-023-04400-3)

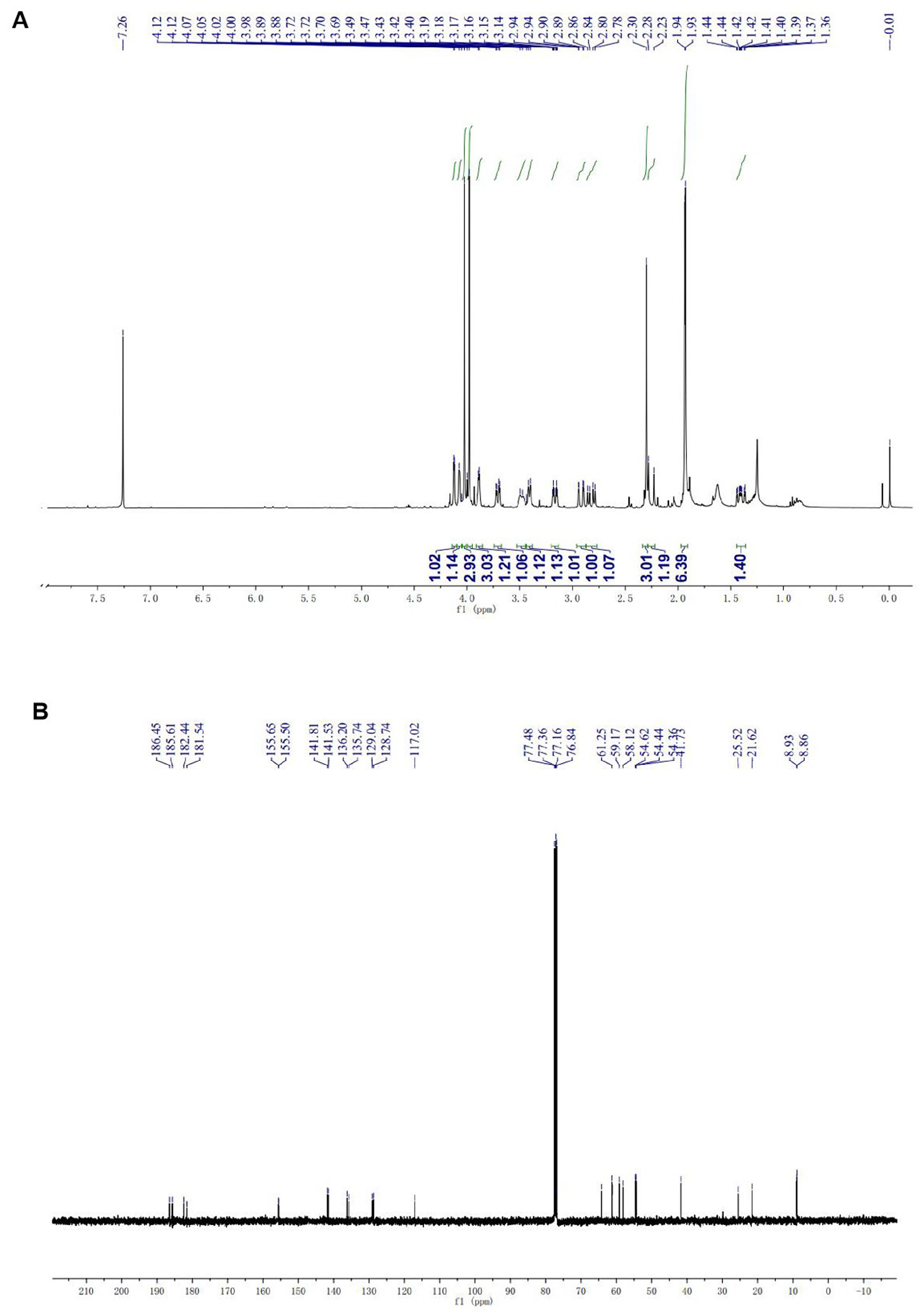

Supplement: Supplementary file 1 — Additional file 1: Fig. S1. NMR spectrum of JorA. (A) 1H NMR spectrum of JorA (400 MHz, CDCl3). (B) 13C NMR spectrum of JorA (100 MHz, CDCl3). [file 12967_2023_4400_MOESM1_ESM.tif]

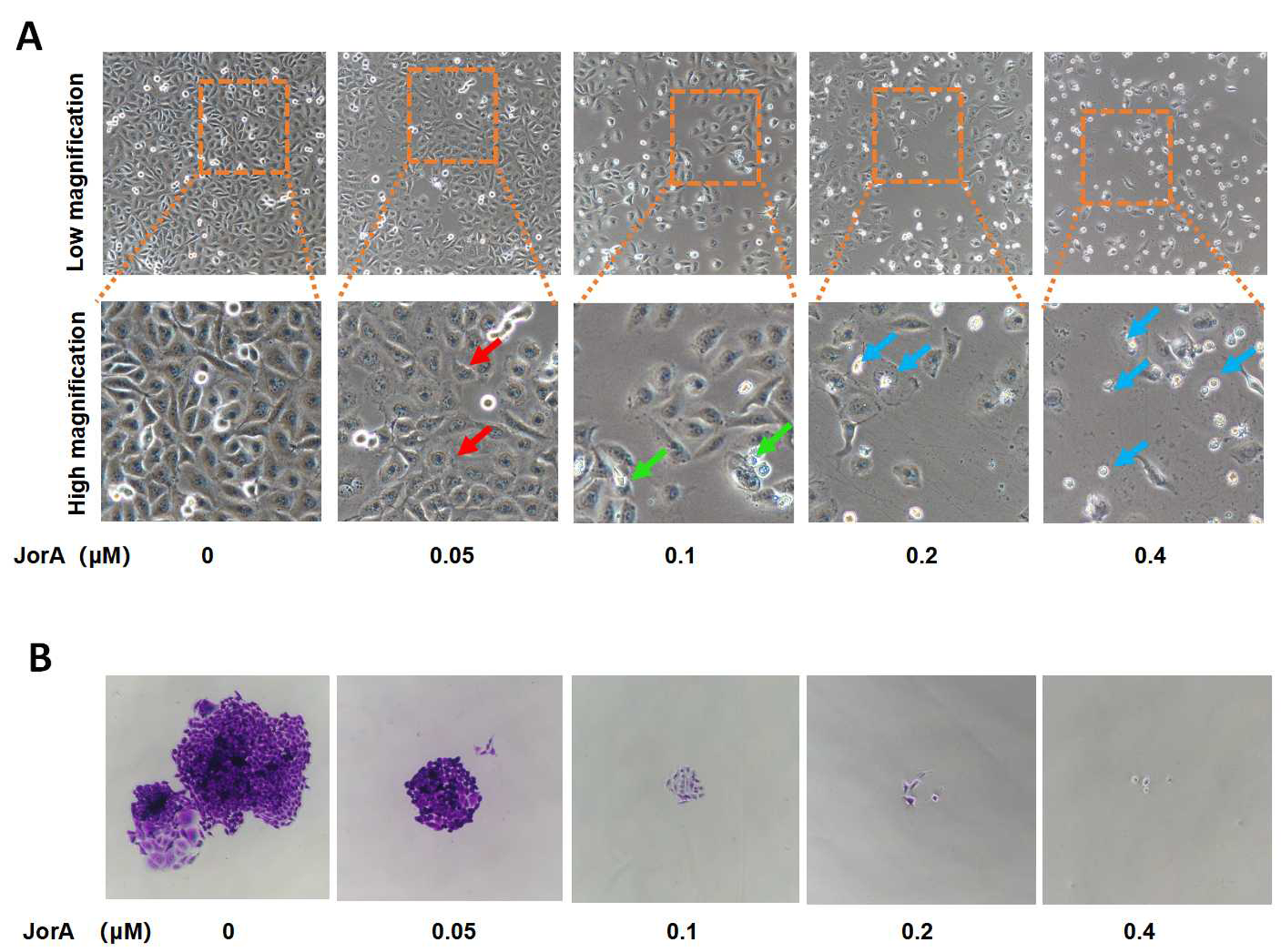

Supplement: Supplementary file 2 — Additional file 2: Fig. S2. JorA inhibited bladder cancer UM-UC-3 cells in vitro. (A) UM-UC-3 cells were treated by 0 − 0.4 μM of JorA for 48 h. Cellular morphology changes were examined under a microscope. Red arrows indicate the UM-UC-3 cells with decreased extensibility, green arrows indicate cells with membrane perforation, blue arrow indicated dead cells. (B) UM-UC-3 cells were incubated with 0 − 0.4 μM of JorA for 14 days. The cells was fixed by 4% PFA and stained with crystal violet. [file 12967_2023_4400_MOESM2_ESM.tif]

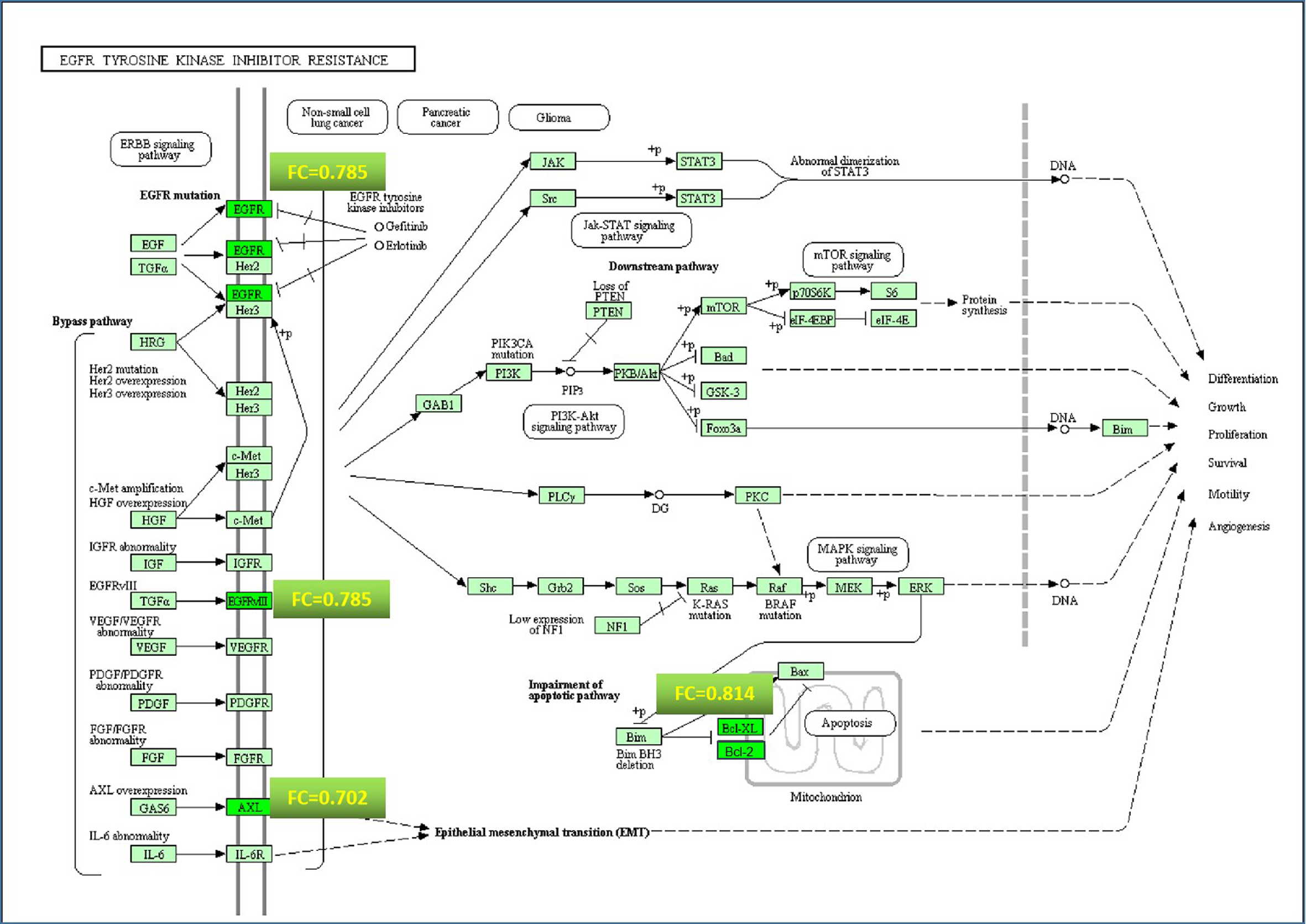

Supplement: Supplementary file 3 — Additional file 3: Fig. S3. KEGG map of JorA down-regulating the key molecules of EGFR tyrosine kinase inhibitor resistance pathway. The KEGG enrichment result of differential proteome indicated that JorA could affect EGFR tyrosine kinase inhibitor resistance pathway by inhibiting EGFR, AXL and Bcl-2. The molecules in the bright green square represent the molecules that are significantly downregulated by JorA, and the fold changes are shown in yellow text. [file 12967_2023_4400_MOESM3_ESM.tif]

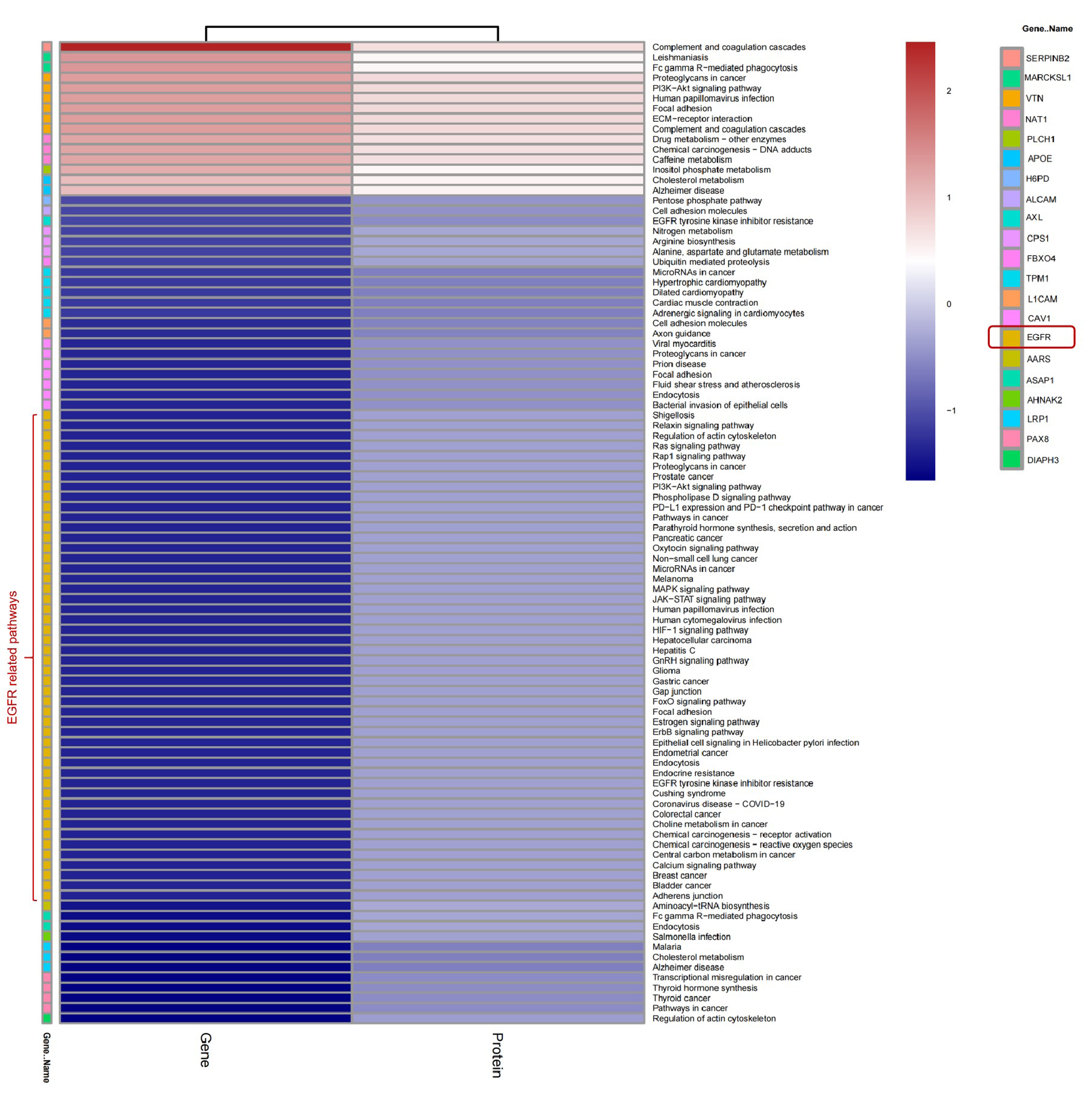

Supplement: Supplementary file 4 — Additional file 4: Fig. S4. JorA mainly targeted EGFR involved pathways. Combined analysis of transcriptome and proteome showed that the EGFR related pathway was dominant in the JorA-induced transcriptome and proteome alteration. [file 12967_2023_4400_MOESM4_ESM.tif]

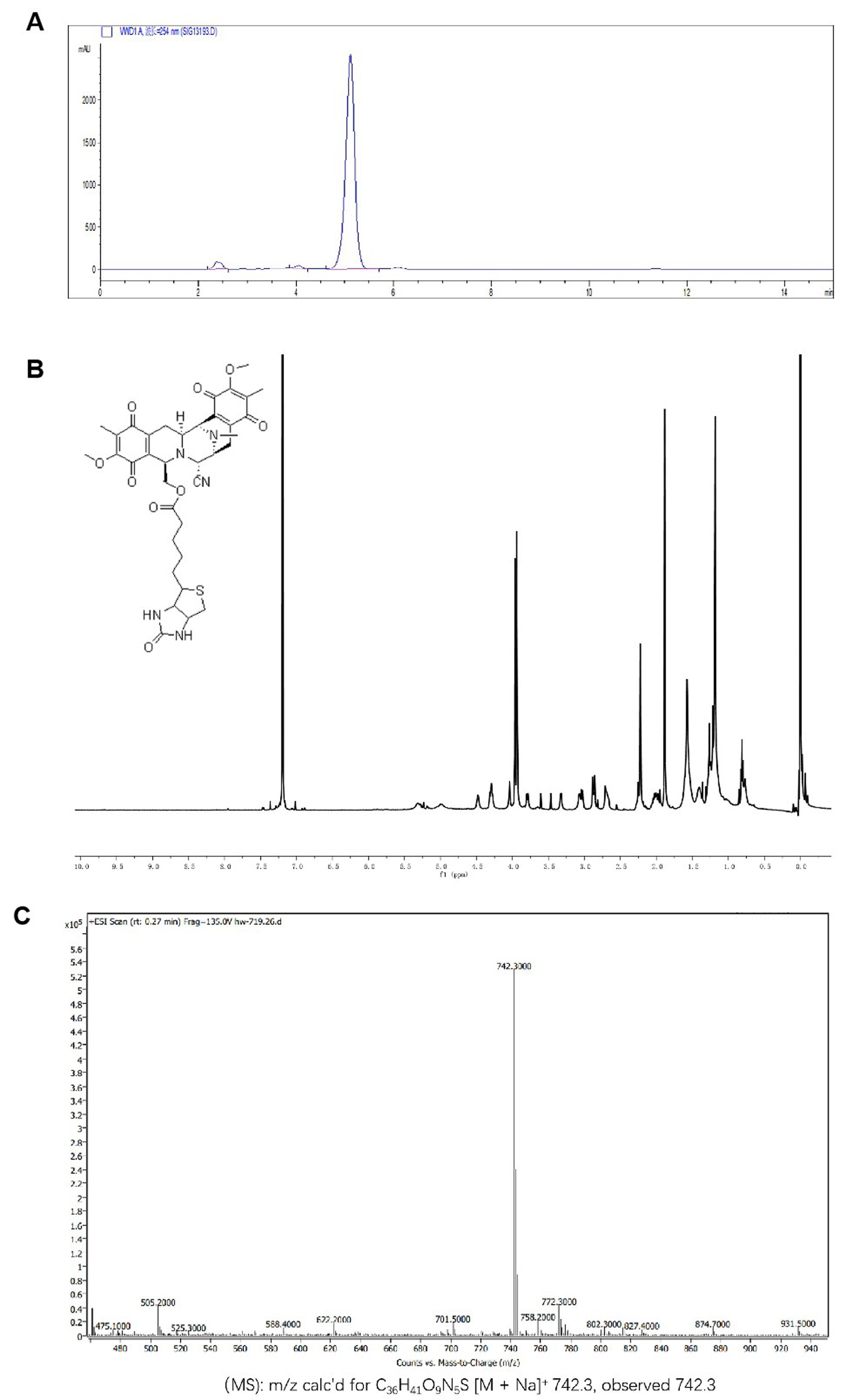

Supplement: Supplementary file 5 — Additional file 5: Fig. S5. Identification of biotin-labeled JorA. (A) HPLC data of biotin-labeled JorA. (B) 1H NMR spectrum of biotin-labeled JorA (400 MHz, CDCl3). (C) MS spectrum of biotin-labeled JorA. [file 12967_2023_4400_MOESM5_ESM.tif]
